# Diagnostic yield of standardized screening for deep venous thrombosis in patients with acute cerebral ischemia and cardiac right-to-left shunt

**DOI:** 10.1186/s42466-025-00396-x

**Published:** 2025-06-06

**Authors:** K.M. Michael, L.P. Pallesen, D.P.O. Kaiser, T. Siepmann, J. Barlinn, A. Sedghi, N. Weiss, M. Weise, S. Werth, K. Barlinn, Volker Puetz

**Affiliations:** 1 Department of Neurology, Medical Faculty and University Hospital Carl Gustav Carus, Technische Universität Dresden, Fetscherstrasse. 74, 01307 Dresden, Germany; 2 Dresden Neurovascular Center, Medical Faculty and University Hospital Carl Gustav Carus, Technische Universität Dresden, Dresden, Germany; 3 Department of Internal Medicine, Division of Angiology, Medical Faculty and University Hospital Carl Gustav Carus, Technische Universität Dresden, Dresden, Germany; 4 Department of Internal Medicine, Medical Faculty and University Hospital Carl Gustav Carus, Technische Universität Dresden, Dresden, Germany; 5 Institute of Neuroradiology, Medical Faculty and University Hospital Carl Gustav Carus, Technische Universität Dresden, Dresden, Germany

**Keywords:** Patent foramen ovale, Deep vein thrombosis, Ultrasound, Ischemic stroke, TIA

## Abstract

**Background:**

Paradoxical embolism is a potential pathophysiology in patients with acute ischemic stroke or transient ischemic attack (TIA) and patent foramen ovale (PFO) or atrial septal defect (ASD). We sought to determine the frequency of deep vein thrombosis (DVT) detection by standardized lower extremity venous compression ultrasound (LE-CUS) in patients with acute cerebral ischemia and cardiac right-to left shunt due to PFO or ASD on transoesophageal echocardiogram (TEE).

**Methods:**

We analysed consecutive patients (01/2015-12/2020) with acute cerebral ischemia and PFO or ASD on TEE, who received DVT screening by LE-CUS per institutional standard. We determined clinical baseline variables including shunt-size categorized as small, medium or large, and analysed the frequency of DVT. We performed multivariable analysis to identify predictors for presence of DVT on LE-CUS.

**Results:**

Among 1564 patients with acute ischemic stroke (*n* = 1326) or TIA (*n* = 238) who received TEE, 390 patients had PFO and 10 patients ASD, of whom 274 were screened for DVT by LE-CUS (153 [55.8%] female, age 64 years [51–76], NIHSS score 4 [1-9.5]). Of these, 55 patients (20.1%) had DVT on LE-CUS. Among patients with DVT, 23 of 76 patients (30.3%) who received LE-CUS within 72 h from admission compared to 32 of 198 patients (16.2%) who received LE-CUS at later time points had presence of DVT (*p* = 0.012). The percentage of patients with DVT tended to be higher among patients with cryptogenic ischemic stroke compared to patients with other stroke etiologies (21.8% [49 of 225] vs. 12.2% [6 of 49]; *p* = 0.168). Presence of DVT was associated with female sex (OR 2.24, 95%CI 1.09–4.62), NIHSS score (OR 1.06, 95%CI 1.03–1.10), Wells score (OR 1.54, 95%CI 1.11–2.13) and shunt size (OR 3.32, 95%CI 1.86–5.91).

**Conclusions:**

Our data suggest a high diagnostic yield (> 20%) of standardized screening for DVT with LE-CUS in patients with acute cerebral ischemia and PFO or ASD. This particularly applies to females, patients with more severe baseline deficits and large right-to-left shunt. These findings may not be generalizable to all patients with PFO or ASD and need prospective validation.

## Background

Stroke is a leading cause of acquired disability and death worldwide [1]. In patients with acute ischemic stroke or transient ischemic attack (TIA), the identification of the underlying aetiology is one of the most relevant tasks provided by stroke-physicians in order to initiate patient-specific secondary prophylaxis. Therefore, extensive diagnostic work-up is part of acute stroke-unit care and routinely includes duplex ultrasound of extra- and intracranial cerebral arteries, monitoring for atrial fibrillation and transthoracic (TTE) and frequently transoesophageal echocardiography (TEE) [2, 3, 4]. Cardiac embolism applies for about 25% of stroke aetiologies with atrial fibrillation being the most frequent entity [5]. Moreover, structural heart diseases like atrial thrombus or infective endocarditis are potential sources for cardiac embolism and can be detected by TEE [3, 6]. 

Patent foramen ovale (PFO) is present in 25–30% of an average healthy population and in up to 50% of patients with cryptogenic stroke [7, 8]. Percutaneous PFO closure is recommended for secondary prevention in patients with cryptogenic embolic stroke and medium to large PFO or accompanying atrial septal aneurysm (ASA) aged 60 years or younger [9]. Paradoxical embolism from deep venous thrombosis (DVT) is a proposed stroke mechanism in these patients [10, 11, 12]. Atrial septal defect (ASD) represents a similar, but less frequent pathway for paradoxical embolism [13]. However, the frequency of DVT in ischemic stroke patients with PFO or ASD is largely unknown. Previous studies describe a DVT-frequency varying between 7% and 20% [14, 15, 12]. Most of these studies utilized MR- or CT-venography instead of universally available and non-invasive techniques [16, 12]. Few studies have investigated the diagnostic yield of routine screening for DVT with lower extremity venous compression ultrasound (LE-CUS) in stroke patients with PFO resulting in DVT-frequencies of 7–8%, with no differences among patients with cryptogenic compared to non-cryptogenic stroke aetiology [14, 17, 15]. 

The aim of our study was to assess the diagnostic yield of standardized surveillance with LE-CUS for DVT in patients with acute cerebral ischemia and PFO or ASD. We further aimed to identify predictors for the presence of DVT in order to streamline diagnostic procedures in the clinical setting.

## Methods

### Study population

We performed a retrospective descriptive cross-sectional study of consecutive adult patients who presented to our tertiary care stroke centre between January 1st, 2015 to December 31st, 2020. Inclusion criteria were a final clinical diagnosis of acute ischemic stroke or TIA, TEE demonstrating PFO or ASD with right to left shunt according to the judgement of the performing cardiologist and screening for DVT with LE-CUS.

Routine diagnostic workup for patients with ischemic stroke or TIA at our centre includes non-contrast computed tomography (NCCT) and CT-angiography (CTA) or magnetic resonance imaging (MRI) including MR-angiography (MRA), screening for atrial fibrillation with Holter ECG for at least 24 h, duplex ultrasound of extra- and intracranial cerebral arteries and TTE. All patients received prophylaxis of DVT which predominantly consisted of treatment with low molecular weight heparin in prophylactic dosage during the study period (Ringleb P [18]). TEE was performed in patients with hitherto etiologically unexplained ischemic stroke or TIA and potential therapeutic implications (e.g., start of anticoagulation). Patients with known or newly detected atrial fibrillation did not routinely receive TEE for further diagnostic workup. During the study period, we performed screening for DVT with LE-CUS in the majority of patients with PFO or ASD per institutional standard regardless of clinical symptoms or laboratory findings suggesting DVT.

### Data collection

We recorded routine clinical parameters from electronic health records. We collected information on age, gender, final clinical diagnosis as categorized into ischemic stroke or TIA, vascular risk factors, baseline and discharge National Institutes of Health Stroke Scale (NIHSS) scores, modified Rankin scale (mRS) score at discharge, the ABCD2 score for patients with TIA, the final stroke etiology as categorized with the Trial of Org 10,172 in Acute Stroke Treatment (TOAST) criteria [19], and acute treatment modalities including intravenous thrombolysis (IVT) and endovascular therapy (EVT). Moreover, we collected potential risk factors and clinical signs and symptoms for DVT if recorded in patient files, as presented in the Wells score, and data to calculate the Risk of Paradoxical Embolism (RoPE) Score based on clinical parameters and imaging reports [20, 21]. Imaging data were noted based on final neuroradiological reports and included information on localization, size and distribution pattern (e.g., lacunar, embolic) of the ischemic lesion, as well as a symptomatic occlusion of an intracranial artery. We further gathered selected laboratory parameters (leucocyte and thrombocyte count, CRP, coagulation parameters, HbA1c, LDL-, HDL- and triglyceride-levels) which were collected during the hospital course.

For comparative multivariable analysis we only included patients who had received LE-CUS. To test for selection bias regarding the performance of LE-CUS we assessed baseline variables (i.e., age, gender, baseline NIHSS score, vascular risk factors) of patients who had PFO or ASD on TEE but did not receive LE-CUS during the hospital course.

### TEE procedure

TEE was performed by an experienced cardiologist according to the German Society of Cardiology Guidelines. Before insertion, TEE probes were covered in lidocaine gel. An additional conscious sedation was achieved, when necessary, by intravenous application of benzodiazepines or propofol. For detection of a possible right to left shunt an agitated GELAFUSAL^®^ solution (4% gelatine in Ringer-Acetate solution) was injected under observation of the left atrium, the interatrial septum and partially the right atrium. PFO was diagnosed if TEE demonstrated right to left shunt with detection of contrast-bubbles in the left atrium spontaneously or after performance of Valsalva maneuveur. Depending on the functional shunt-volume depending on the amount of contrast-bubbles passing from right to left atrium, PFO-shunt size was visually categorized into small, medium, or large according to the discretion of the cardiologist. Additional ASA was diagnosed in patients with hypermobility of the interatrial septum resulting in septal excursion from the midline into the right or left atrium > 10 mm [22, 23]. Patients with ASD were only included if right to left shunt was proven.

### Screening for DVT with LE-CUS

Among patients with PFO or ASD, most patients underwent LE-CUS by vascular specialists experienced in complete compression ultrasound using a standardized protocol before discharge [24, 25]. Screening for LE-CUS was performed regardless of clinical signs and symptoms suggesting the presence of DVT per institutional standard.

### Outcome measures

Our primary outcome measure was the detection of DVT or muscular vein thrombosis with contact to the deep venous system with the absence of signs of a past thrombosis, superficial thrombosis, or thrombophlebitis. This outcome is summarized as DVT throughout the further manuscript. Secondary outcome measures were favourable functional outcome at discharge defined as modified Rankin Scale (mRS) scores of 0 to 2 and in-hospital mortality (mRS 6).

### Statistical analysis

Continuous variables are presented as mean (± standard deviation, ±SD) and median (interquartile range, IQR). Non-continuous variables are described as frequency in percent. Between-group comparisons were performed using the Pearson chi-square test, Fisher’s exact test and Two-sample Wilcoxon rank-sum (Mann-Whitney) test when applicable. Multivariable analysis was determined using stepwise logistic regression to calculate predictors for the presence of DVT among the recorded variables. A priori selected variables known to be associated with DVT (female sex, cancer, Wells score) and further variables (age, CRP-level and leukocyte count, active cancer) which were associated with DVT in univariable analysis at a p-level < 0.10 were chosen as covariates in the multivariable regression model. To test for selection bias, the baseline variables of the study population were compared with the group of patients with PFO who did not receive LE-CUS during the hospital course using the Mann-Whitney-U-Test. Moreover, we performed sensitivity analyses to analyse: (1) the frequency of patients with DVT among screened patients with cryptogenic ischemic stroke compared to other TOAST etiologies; and (2) the frequency of patients with DVT among patients who received screening with LE-CUS within 72 h from admission compared to patients who received screening with LE-CUS at later time-points during the hospital course. Significance level was set at *p* < 0.05. All analyses were computed with STATA 16.1 (StataCorp LLC) and IBM SPSS Statistics Version 27 (IBM Corp.).

## Results

### Patients

**Fig. 1:**
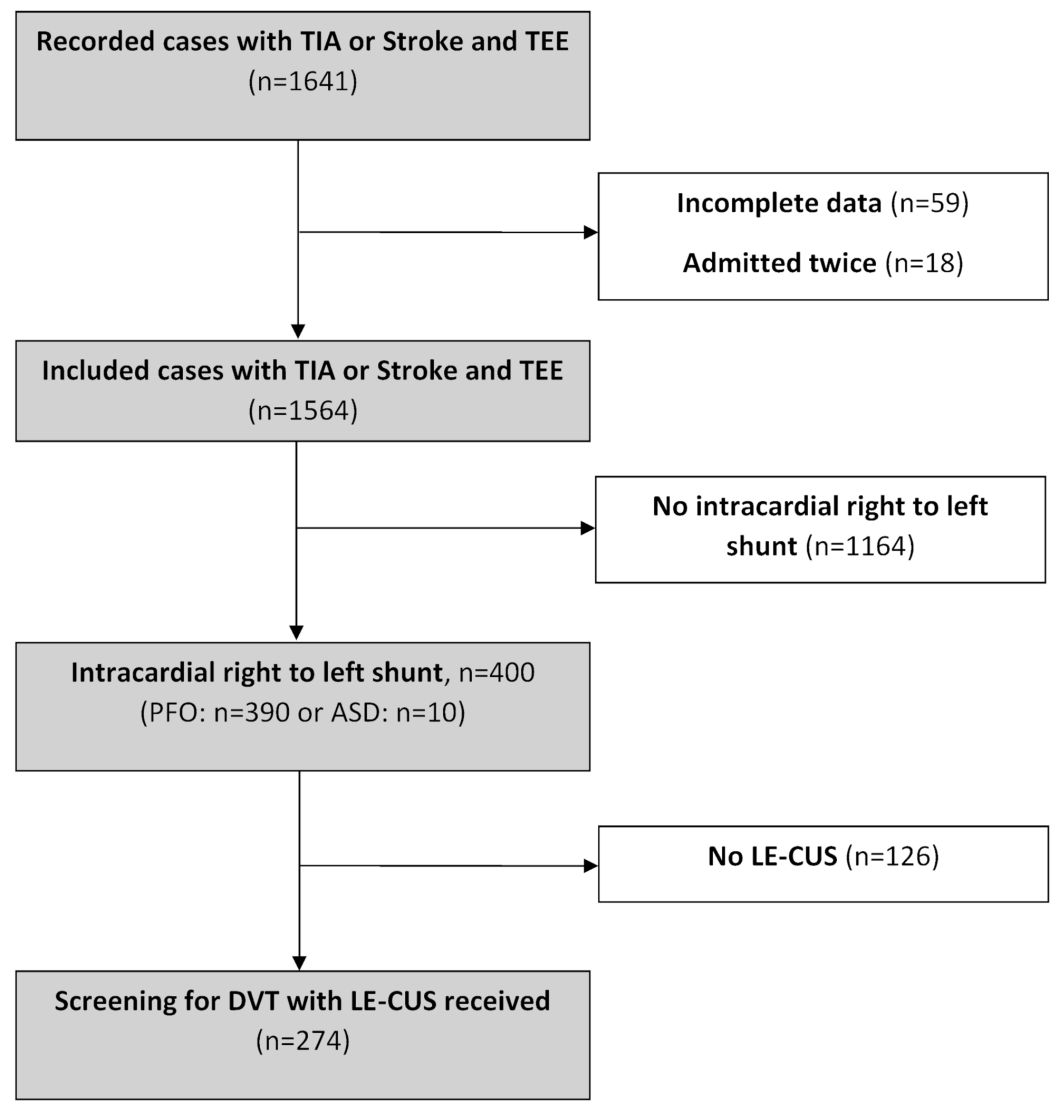
Flow diagram

Among 1641 recorded cases of patients presenting with TIA (*n* = 260) or ischemic stroke (*n* = 1381) who received TEE during their hospital course, we excluded 77 cases due to incomplete data (*n* = 59) or if patients were admitted twice during the study period (*n* = 18). Among the resulting 1564 patients, 390 (24.9%) patients had PFO and 10 (0.6%) patients had ASD with right to left shunt, of whom 274 patients were screened for DVT with LE-CUS (Fig. 1).

Compared to patients receiving LE-CUS, patients with PFO or ASD who were not screened for DVT tended to be older and were less likely to be female, were more likely to have suffered TIA rather than ischemic stroke, were more likely to have known diabetes, arterial hypertension, coronary artery disease, or peripheral vascular disease, and were less likely to have cryptogenic ischemic stroke rather than other stroke etiologies as defined by TOAST-criteria (Table 1).

**Table 1:** Comparison of patients with intracardial right to left shunt due to PFO or ASD on TEE, depending on performance of LE-CUS

Parameter	All patients	Received LE-CUS	No LE-CUS	*p*-value
Number, [n] (%)	400	274 (68.5)	126 (31.5)	-
Age (years), mean (IQR)		62.51 (51–76)	66.12 (58–77)	0.054
Gender female, (%)	163 (40.75)	121 (44.1)	42 (33.3)	0.041
TIA, (%)	48 (12)	21 (7.7)	27 (21.4)	0.000
Ischemic stroke, (%)	352 (88)	253 (92.3)	99 (78.6)	0.000
Baseline NIHSS score, median (IQR)	3 (5–13)	4 (1–9)	3 (1–6)	0.383
**Vascular risk-factors**				
Art. hypertension (%)	283 (70.75)	184 (67.2)	99 (78.6)	0.020
Diabetes (%)	98 (24.5)	55 (20)	43 (34.1)	0.002
Hyperlipidemia (%)	255 (63.75)	169 (61.7)	86 (68.3)	0.204
Peripheral artery disease	9 (2.25)	5 (1.8)	4 (3.2)	0.471
Coronary artery disease	48 (12)	21 (7.7)	27 (21.4)	0.000
Current smoker	83 (20.75)	55 (20.1)	28 (22.2)	0.622
Current cancer	25 (6.25)	15 (5.5)	10 (7.9)	0.377
**TOAST criteria**				
Large-artery atherosclerosis (%)	31 (7.75)	17 (6.2)	14 (11.1)	0.002
Cardioembolic stroke (%)	33 (8.25)	15 (5.5)	18 (14.3)	
Small vessel disease (%)	25 (6.25)	14 (5.1)	11 (8.7)	
Other determined source (%)	5 (1.25)	3 (1.1)	2 (1.6)	
Cryptogenic stroke (%)	306 (76.5)	225 (82.1)	81 (64.3)	

IQR indicates interquartile range; TIA, transient ischemic attack; NIHSS, National Institute of Health Stroke Scale; PFO, patent foramen ovale; ASD, atrial septal defect

Baseline data of 274 patients with PFO (*n* = 265) or ASD (*n* = 9) who received LE-CUS during the hospital course are summarized in Table 2. The median time from patient admission to examination with LE-CUS was 5 days (IQR 3–7). Overall, 253 patients (92.3%) had a final diagnosis of ischemic stroke, 121 (44.2%) were female, the mean (± SD) age was 62.5 years (± 16.0), the median baseline NIHSS score was 4 (1-9.5), and the median Wells score was 0 (0–1). The shunt size due to PFO was categorized as small in 175 patients (71.7%), moderate in 57 patients (23.4%) and large in 12 patients (4.9%). PFO and additional ASA was diagnosed in 55 of 274 patients (20.1%). Regarding acute treatment, overall, 69 patients (25.2%) received IVT, 39 (14.2%) patients received EVT, and 23 patients (8.4%) received combined IVT and EVT.

**Table 2:** Clinical baseline characteristics and diagnostic test results in patients with intracardial right to left shunt due to PFO or ASD who received LE-CUS

Parameter	All Patients	DVT	no DVT	*p*-value
Number, n (%)	274*	55 (20.1)	219 (79.9)	-
Age (years), mean (± SD)	62.5 (± 16.0)	65.9 (± 14.6)	61.7 (± 16.3)	0.086
Gender female, n (%)	121 (44.2)	34 (61.8)	87 (39.7)	0.003
Time from admission to LE-CUS (days), median (IQR)	5 (3–7)	4 (1–8)	5 (4–7)	0.277
Stroke (vs. TIA), (n (%)	253 (92.3)	53 (96.4)	200 (91.3)	0.209
**Vascular Risk factors**,** n (%)**				
Diabetes mellitus	55 (20.1)	8 (14.5)	47 (21.5)	0.252
Hypertension	184 (67.2)	41 (74.5)	143 (65.3)	0.192
Hyperlipidemia	169 (61.7)	28 (50.9)	141 (64.4)	0.066
Current smoking	55 (20.1)	7 (12.7)	48 (21.9)	0.128
Coronary artery disease	21 (7.7)	1 (1.8)	20 (9.1)	0.068
Peripheral artery diseases	5 (1.8)	0 (0)	5 (2.3)	0.587
Prior Stroke	93 (33.9)	14 (25.5)	79 (36.1)	0.137
Prior TIA	18 (6.6)	3 (5.5)	15 (6.8)	0.709
Active cancer	26 (9.5)	6 (10.9)	20 (9.1)	0.688
**Index stroke data**				
Baseline NIHSS score, median (IQR)	4 (1-9.5)	10 (2–24)	2 (1–6)	0.000
IVT, n (%)	69 (25.2)	16 (29.1)	53 (24.2)	0.455
EVT, n (%)	39 (14.2)	17 (30.9)	22 (10.0)	0.000
Combined therapy, n (%)	23 (8.4)	9 (16.4)	14 (6.4)	0.017
**Scores**,** median (IQR)**				
Wells score	0 (0–1)	1 (0–2)	0 (0–1)	0.001
RoPE score	5 (4–7)	4 (4–6)	5 (4–7)	0.424
**Baseline laboratory results**,** mean (± SD)**				
Leucocyte count (10^3^/µl)	8.8 (± 3.4)	10.0 (± 4.2)	8.5 (± 3.1)	0.020
CRP (mg/L)	10.7 (± 29.6)	19.6 (± 38.9)	8.4 (± 26.3)	0.001
HbA1_C_ (mmol/mol)	38.8 (± 10.3)	37.6 (± 4.9)	39.1 (± 11.2)	0.554
**PFO associated data**,** n (%)**	244 (89.1)	51 (92.7)	193 (88.1)	
Right to left shunt, large	12 (4.9)	7 (13.7)	5 (2.6)	0.000
Right to left shunt, moderate	57 (23.4)	21 (41.2)	36 (18.7)	
Right to left shunt, small	175 (71.7)	23 (45.1)	152 (78.8)	
ASA	55 (20.1)	14 (25.5)	41 (18.7)	0.265
**Acute treatment of DVT**,** n (%)**				
LMWH	-	32 (58.2)	-	-
i.v. Heparin	-	7 (12.7)	-	-
DOAC	-	14 (25.5)	-	-
Vena cava filter implantation only	-	2 (3.6)	-	-
Vena cava filter implantation with additional anticoagulation (e.g., LMWH or Heparin)	-	5 (9.1)	-	-
**TOAST criteria**				
Large-artery atherosclerosis (%)	17 (6.2)	1 (1.8)	16 (7.3)	0.033
Cardioembolic stroke (%)	15 (5.5)	3 (5.5)	12 (5.5)	
Small vessel disease (%)	14 (5.1)	0 (0)	14 (6.4)	
Other determined source (%)	3 (1.1)	2 (3.6)	1 (0.5)	
Cryptogenic stroke (%)	225 (82.1)	49 (89.1)	176 (80.4)	
**Functional outcome at discharge**,** n (%)**				
Favourable (mRS Score 0–2)	208 (75.9)	27 (49.1)	181 (82.6)	0.000
Deceased	11 (4.0)	7 (12.7)	4 (1.8)	0.000

ASD indicates atrial septal defect; PFO, patent foramen ovale; SD, standard deviation; LE-CUS, lower extremity venous compression ultrasound; IQR, interquartile range; TIA, transient ischemic attack; IVT, intravenous thrombolysis therapy; EVT, endovascular thrombectomy; NIHSS, National Institute of Health Stroke Scale; RoPE Score, Risk of Paradoxical Embolism Score; CRP, C reactive protein; LMWH, low molecular weight heparin; i.v., intravenous; DOAC, direct oral anticoagulants; mRS, modified Rankin Scale. *Thereof patients with PFO: 265; with ASD: 9

At discharge, 208 (75.9%) patients had a favourable functional outcome, 55 (20.1%) patients had an unfavourable outcome, and 11 (4.0%) patients were deceased.

### Detection of DVT with LE-CUS

LE-CUS detected DVT (*n* = 47) or muscular vein thrombosis with contact to the deep venous system (*n* = 8) in 55 of 274 patients (20.1%). The dimension and location was detected as follows: 30 of 55 patients (54.5%) had a 1-level thrombosis, 11 patients (20.0%) a 2-level thrombosis, 10 patients (18.1%) a 3-level thrombosis and 4 patients (7.3%) a 4-level thrombosis. Among the 1-level thromboses, 8 (26.6%) were isolated muscular vein thromboses with documented connection to the deep venous system, 20 (66.6%) were located in the calves and 2 (6.6%) in the thigh region. The majority (10 of 11) of the 2-level thromboses were located distally (calves and popliteal region), all 3-level thromboses extended from the calves to the thigh region.

Clinical baseline data and results of diagnostic tests according to presence of DVT are summarized in Table 2. Compared to patients without DVT, DVT-patients were more likely to be female, had higher baseline NIHSS score, larger shunt size, higher Wells score and were more likely to have cryptogenic ischemic stroke rather than other stroke etiologies as defined by TOAST-criteria. Additionally, patients with DVT more often received EVT or combined IVT and EVT. At discharge, patients with DVT were less likely to have an independent functional outcome and more likely to be deceased.

Among patients with DVT, clinical signs and symptoms (pain or tenderness along deep venous system [*n* = 5], limb oedema [*n* = 4], difference in circumference of the lower legs [*n* = 2]) were present in 11 of 55 patients (20%) only. The median Wells Score was low in patients with and without DVT (median [IQR] 1 [0–2] vs. 0 [0–1]; *p* = 0.001). Patients who had DVT were primarily treated with LMWH in 32 patients (58.2%), intravenous heparin in 7 patients (12.7%), inferior vena cava filter implantation in 7 patients (12.7%) (5 of them with combined vena cava filter and anticoagulation) or direct oral anticoagulants (DOAC) in 14 patients (25.5%). Among patients with DVT, 19 (34.5%) were also diagnosed with pulmonary embolism. Imaging to detect pulmonary embolism was not routinely performed in patients with DVT.

### Predictors for presence of DVT on LE-CUS

In multivariable analysis, female gender, higher Wells score, larger shunt-size and higher baseline NIHSS score were associated with the detection of DVT on LE-CUS (Table 3). The RoPE-score was not associated with the presence of DVT in multivariable analysis (*p* = 0.424).

**Table 3:** Multivariable analysis for detection of DVT with LE-CUS in patients with PFO or ASD

Variable	Comparison	Adjusted Odds Ratio (95%CI)
Gender, female	yes vs. no	2.24 (1.09–4.62)
Baseline NIHSS score	per point increase	1.06 (1.03–1.10)
Well score	per 1-unit increase	1.54 (1.11–2.13)
Size of shunt	categorized as small, moderate, or large	3.32 (1.86–5.91)

NIHSS indicates National Institute of Health Stroke Scale; CI, confidence interval; DVT, deep vein thrombosis; LE-CUS, lower extremity venous compression ultrasound; PFO, patent foramen ovale; ASD, atrial septal defect

TIA indicates transient ischemic attack; TEE, transoesophageal echocardiography; LE-CUS, lower extremity venous compression ultrasound; PFO, patent foramen ovale; ASD, atrial septal defect; DVT, deep vein thrombosis

### Sensitivity analyses

The percentage of patients with DVT tended to be higher among patients with cryptogenic ischemic stroke compared to patients with other stroke etiologies as defined by TOAST-criteria (21.8% [49 of 225] vs. 12.2% [6 of 49]; *p* = 0.168). Regarding the time-point of screening for DVT, 23 of 76 patients (30.3%) who received screening with LE-CUS within 72 h from admission compared to 32 of 198 patients (16.2%) who received LE-CUS at later time points during the hospital course had presence of DVT (*p* = 0.012).

## Discussion

Our data suggest a high (> 20%) diagnostic yield of standardized screening for DVT with LE-CUS in patients with acute cerebral ischemia and intracardial right to left shunt. This particularly applies to females, patients with more severe baseline deficits and large right-to-left shunt on TEE. The low Wells score in our study-population indicates that only few of these patients had clinical signs and symptoms indicative of DVT. Moreover, the frequency of DVT tended to be higher among patients with cryptogenic ischemic stroke compared to patients with other TOAST categories.

Corroborating the latter finding, patients who did not receive LE-CUS were less likely to have cryptogenic ischemic stroke compared to patients with DVT screening (64,3% vs. 82.1%). Moreover, among patients who received screening with LE-CUS, patients with DVT were more likely to have cryptogenic ischemic stroke compared to patients without DVT (89.1% vs. 80.4%). As the TOAST etiology was not used as a selection criterion for screening with LE-CUS, reasons for this are speculative, but may point to patients with cryptogenic stroke being a target population with highest DVT-frequency in future studies.

From a clinical point of view, diagnosis of DVT has immediate therapeutic implications as patients require anticoagulation with low molecular weight heparin (LMWH) or oral anticoagulation to prevent pulmonary embolism or stroke recurrence [26, 27]. This can pose clinicians to a therapeutic dilemma as anticoagulation may be contraindicated in patients with acute ischemic stroke due to increased bleeding risk. Consequently, 7 (12.7%) of our patients received reversible inferior vena cava filter implantation during the hospital course to avoid pulmonary embolism and early stroke recurrence. Further studies should focus on balancing the cerebral bleeding risk versus potentially insufficient protection against pulmonary embolism and recurrent stroke due to paradoxical embolism [28]. Moreover, the detection of DVT might contribute to treatment decision making whether to recommend PFO-closure (i.e., argue in favour of PFO-closure) in patients aged 18–60 years with unlikely PFO-related stroke according to the PASCAL classification, or to recommend PFO-closure in patients older than 60 years [9]. 

Lower extremity compression ultrasound is non-invasive and can be performed as a bedside examination for the detection of DVT with high diagnostic accuracy when performed by an experienced examiner. The diagnostic yield of LE-CUS in stroke patients with PFO has only been analysed in few studies where the overall frequency of lower extremity DVT was between 7 to 8% [14, 17, 15]. Further studies utilized either invasive (venography) or more costly and less universally available techniques (CT venography or MR venography) [16, 15, 12]. The percentage of patients with lower extremity DVT in our study is high as compared to other studies with DVT frequencies of < 10% in similar populations [14]. However, the overall DVT frequency in patients with cryptogenic ischemic stroke who have PFO varied between 7 and 27% in a recent review [12]. The screening of stroke patients with MR-venography 7 to 14 days after ischemic stroke yielded an overall venous thromboembolism rate of 40% in a general stroke population, with 18% proximal deep vein thrombosis [16]. However, VTE was predominantly a complication of ischemic stroke rather than potential aetiology, and associated with a low Barthel-Index and non-ambulatory status in this study. Of note, whereas the Wells score was associated with the presence of DVT in our study, the overall Wells score was low and does not seem to be sufficient to reliably exclude stroke patients with PFO who do not require DVT screening [29]. This result is in contrast to a recent paper which demonstrated higher Wells scores in patients with PFO and DVT, and indicated that the Wells score seems to be suitable to guide additional examinations (i.e., to perform LE-CUS) for screening of DVT in patients with cerebral ischemia and concomitant PFO [14]. 

Apart from the Wells score, female gender, higher baseline NIHSS score, and larger shunt-size were independently associated with the detection of DVT with LE-CUS in our study. The association of larger shunt size and higher NIHSS scores with DVT seems pathophysiological valid as venous thrombi may be more likely to cause paradoxical embolism with resulting intracranial large vessel occlusion in this scenario. However, these assumptions remain to be confirmed [30]. Of note, the frequency of patients who received EVT or combined IVT and EVT was higher among patients with DVT in our study. Moreover, patients with DVT were less likely to have a favourable functional outcome and more likely to be deceased. It needs to be determined if worse outcomes are mainly due to the DVT itself or preferentially caused by the more severe baseline stroke symptoms in these patients.

Our data originate from a large cohort of consecutive patients. However, since not all patients with acute cerebral ischemia and PFO or ASD underwent TEE and subsequent LE-CUS, our findings underlie a risk of selection bias. However, as the overall majority of patients with PFO or ASD was screened during the study period regardless of clinical signs and symptoms suggesting DVT per institutional routine, and as the overall Wells score was low in our study cohort, our findings may be representative for a real-world stroke population. Whereas the Wells score might be valid in the preclinical setting, patient history and clinical findings may not reliably exclude DVT in stroke patients with PFO [31, 29, 32]. D-dimer levels were not routinely assessed in our cohort, which may aid the identification of patients who are unlikely to have DVT [31]. However, D-dimer is frequently elevated in ischemic stroke patients, whereby its diagnostic performance to exclude DVT may be limited [33]. Moreover, we cannot exclude that the presence of DVT was a complication of acute ischemic stroke rather than potential stroke aetiology in patients with PFO or ASD. Our data therefore do not imply causality. However, patients routinely received guideline-based DVT prophylaxis during the hospital course and the time-delay from admission to performance of LE-CUS was short (median 4 days in patients with and 5 days in patients without DVT) in our cohort. Moreover, the percentage of patients with DVT was higher among patients who received LE-CUS with 72 h from admission compared to patients who received LE-CUS at later time-points during the hospital course, which may argue that DVT was a likely– though not proven - etiology of the index stroke due to paradoxical embolism rather than a secondary stroke complication. While detailed cardiovascular phenotyping with highly standardized assessment in conjunction with a large sample size support internal validity of our observation, a prospective multicentric investigation is warranted to test its generalizability.

## Conclusions

Our study indicates a high diagnostic yield of standardized screening with LE-CUS for the detection of DVT in ischemic stroke patients with intracardial right to left shunt due to PFO or ASD. Factors associated with DVT were female gender, higher baseline NIHSS score, larger right-to-left shunt, and higher Wells score. Our results support further prospective evaluation and validation in a multicentric patient cohort.

## Data Availability

The datasets used and/or analysed during the current study are available from the corresponding author on reasonable request.

## Abbreviations

TIA: Transient ischemic attack
PFO: Patent foramen ovale
ASD: Atrial septal defect
DVT: Deep venous thrombosis
LE-CUS: Lower extremity venous compression ultrasound
TTE: Transthoracic echocardiography
TEE: Transoesophageal echocardiography
ASA: Atrial septal aneurysm
NCCT: Non-contrast computed tomography
CTA: CT-angiography
MRI: Magnetic resonance imaging
NIHSS: National Institutes of Health Stroke
mRS: Modified Rankin Scale
