# Correction: Diagnostic yield of standardized screening for deep venous thrombosis in patients with acute cerebral ischemia and cardiac right-to-left shunt

**DOI:** 10.1186/s42466-025-00402-2

**Published:** 2025-06-23

**Authors:** K.M. Michael, L.P. Pallesen, D.P.O. Kaiser, T. Siepmann, J. Barlinn, A. Sedghi, N. Weiss, M. Weise, S. Werth, K. Barlinn, Volker Puetz

**Affiliations:** 1 Department of Neurology, Medical Faculty and University Hospital Carl Gustav Carus, Technische Universität Dresden, Fetscherstrasse. 74, 01307 Dresden, Germany; 2 Dresden Neurovascular Center, Medical Faculty and University Hospital Carl Gustav Carus, Technische Universität Dresden, Dresden, Germany; 3 Department of Internal Medicine, Division of Angiology, Medical Faculty and University Hospital Carl Gustav Carus, Technische Universität Dresden, Dresden, Germany; 4 Department of Internal Medicine, Medical Faculty and University Hospital Carl Gustav Carus, Technische Universität Dresden, Dresden, Germany; 5 Institute of Neuroradiology, Medical Faculty and University Hospital Carl Gustav Carus, Technische Universität Dresden, Dresden, Germany

**Correction: Neurological Research and Practice (2025) 7:38**

10.1186/s42466-025-00396-x

In the original article, the author name D.P.O. Kaiser was incorrectly given as ‘D.A. Kaiser’. The original article has been corrected.

